# Minimally Invasive Therapeutic Drug Monitoring of Immunosuppressants in Children with Kidney Diseases: Validation of Fingerstick Sampling Using LC-MS/MS

**DOI:** 10.3390/ph19040630

**Published:** 2026-04-16

**Authors:** Marika Ishii, Jun Aoyagi, Natsuka Kimura, Masanori Kurosaki, Tomomi Maru, Kazuya Tanimoto, Mitsuaki Yoshino, Takane Ito, Takahiro Kanai, Hitoshi Osaka, Ryozo Nagai, Kenichi Aizawa

**Affiliations:** 1 Department of Pediatrics, Jichi Medical University, 3311-1 Yakushiji, Shimotsuke 329-0498, Tochigi, Japan; 2 Department of Translational Research, Clinical Research Center, Jichi Medical University Hospital, 3311-1 Yakushiji, Shimotsuke 329-0498, Tochigi, Japan; 3 Jichi Medical University, 3311-1 Yakushiji, Shimotsuke 329-0498, Tochigi, Japan; 4 Clinical Pharmacology Center, Jichi Medical University Hospital, 3311-1 Yakushiji, Shimotsuke 329-0498, Tochigi, Japan

**Keywords:** pediatric immunosuppressive therapy, therapeutic drug monitoring (TDM), fingerstick blood sampling, microsampling, LC-MS/MS, mycophenolic acid, tacrolimus, cyclosporine A, home-based monitoring

## Abstract

**Background/Objectives**: Therapeutic drug monitoring (TDM) of immunosuppressants is essential in treating pediatric kidney diseases; however, repeated venipuncture is burdensome in children. We evaluated whether minimally invasive fingerstick capillary sampling combined with liquid chromatography–tandem mass spectrometry (LC-MS/MS) provides results analytically comparable to those of conventional venous sampling. **Methods**: Capillary whole blood (2.8 µL) was collected via fingersticks from pediatric patients receiving mycophenolate mofetil, with or without tacrolimus (TAC) or cyclosporine A (CsA). Drug concentrations were quantified using a previously validated simultaneous LC-MS/MS method and compared with conventional venous sampling using linear regression and Bland–Altman analyses. **Results**: Seventy-four paired samples from 21 patients were analyzed. Strong correlations were observed between capillary and venous samples for mycophenolic acid (MPA), TAC, and CsA (R^2^ > 0.90). Hematocrit correction improved agreement for MPA. Bland–Altman analyses demonstrated acceptable bias across analytes. **Conclusions**: Fingerstick-based microvolume sampling combined with LC-MS/MS provides analytically reliable immunosuppressant quantification in pediatric patients. Although larger clinical validation is required, this minimally invasive approach may reduce procedural burden and may support future outpatient or home-based TDM strategies.

## 1. Introduction

Therapeutic drug monitoring (TDM) is crucial for optimizing immunosuppressive therapy in pediatric patients with nephrotic syndrome, lupus nephritis, and post-renal transplantation, all of which require long-term pharmacological immunosuppression [1,2]. Achieving appropriate drug exposure is essential to prevent relapse, rejection, or adverse drug reactions. The key agents used in these conditions—mycophenolic acid (MPA), tacrolimus (TAC), and cyclosporine A (CsA)—exhibit substantial pharmacokinetic variability and narrow therapeutic indices necessitate individualized dosing strategies supported by accurate TDM [3,4].

The gold standard for evaluating MPA exposure involves estimating the area under the concentration–time curve from 0 to 12 h (AUC_0–12_); however, this approach presents significant practical challenges in children [2,5,6]. Repeated venipuncture is often hindered by small-caliber veins, a high risk of vein collapse after a single puncture, and limited tolerance for repeated needle insertion. Even with the use of an indwelling catheter, sufficient blood reflux may not be achieved due to the small vessel diameter. As a result, scheduled sampling can be missed, compromising data accuracy. Moreover, older children and adolescents often require frequent outpatient visits solely for TDM, resulting in school absenteeism and disruption of daily life. These burdens underscore an unmet need for less invasive, patient-centered monitoring strategies in children receiving immunosuppressive therapy.

To address these limitations, minimally invasive microsampling techniques are needed. Approaches such as dried blood spot (DBS) sampling and volumetric absorptive microsampling (VAMS) enable reliable bioanalysis using extremely small volumes of capillary blood and have been widely investigated for pharmacokinetic studies and TDM [7,8]. Recent advances in microsampling technologies have further expanded their potential clinical applications, particularly in pediatric and transplant populations, where reducing sampling burden is especially important [9,10].

Recent clinical studies have also demonstrated the feasibility of microsampling-based monitoring in pediatric kidney diseases. For example, DBS sampling provides reliable TDM of immunosuppressive agents in children with immune-mediated glomerulopathies and kidney transplantation, showing good agreement with conventional venous sampling [11].

In pediatric settings, fingerstick-based capillary sampling represents a particularly attractive approach because it can reduce procedural burden while facilitating repeated sampling in outpatient environments [12]. Inoue et al. compared venous blood samples with fingertip capillary blood collected using the Capillary Cup^®^ (Japan Medical Leaf Co., Ltd., Tokyo, Japan) via a capillary microsampling approach and demonstrated good correlations in hematological and routine biochemical parameters. Furthermore, no adverse events, such as difficulty in hemostasis or accidental puncture, were reported with the use of the Capillary Cup^®^ in their study [12]. However, its clinical applicability and analytical reliability, particularly in the context of multi-agent immunosuppressive regimens, remain to be thoroughly validated.

At the same time, recent advances in the sensitivity and selectivity of liquid chromatography–tandem mass spectrometry (LC-MS/MS) have enabled simultaneous quantification of multiple immunosuppressive agents from minimal blood volumes, thereby reducing the total blood volume required compared with separate individual analyses [13]. The combination of fingerstick-based microsampling and high-sensitivity LC-MS/MS therefore holds great promise for addressing the unmet clinical needs of pediatric patients by offering a practical and less burdensome solution for accurate TDM. This approach may ultimately enable outpatient or home-based sampling and may support more accessible and individualized pharmacotherapy in pediatric immunosuppressive treatment.

Importantly, although our previous investigation of micro-volume LC-MS/MS-based therapeutic drug monitoring in adult transplant recipients demonstrated the analytical feasibility of ultra-low-volume sampling, whether this approach can be reliably implemented in pediatric clinical practice—where physiological, procedural, and ethical constraints differ substantially—remains insufficiently explored [14].

Although microsampling approaches such as VAMS and DBS have been investigated in pediatric populations, most studies have focused on single analytes or specific disease settings [2,15]. Consequently, comprehensive validation of simultaneous quantification of multiple immunosuppressants using ultra-low-volume capillary whole blood in children remains limited.

Therefore, in the present study we applied a previously developed LC–MS/MS method capable of simultaneously quantifying multiple key immunosuppressive agents from 2.8 µL of whole blood (hereafter referred to as the microvolume method; MM) to fingerstick-derived capillary samples obtained from pediatric patients [13]. The analytical validity and practical feasibility of this approach were evaluated by comparing drug concentrations measured in capillary samples with those obtained from conventional venous blood samples.

## 2. Results

A total of 22 patients were enrolled in this study. One patient was excluded from the MPA analysis because the measured MPA concentration was below the detection limit; thus, 21 patients were included in the final analysis. Patient demographics are summarized in Table 1. Among these 22 patients, 6 were receiving concomitant TAC, and 5 were receiving concomitant CsA. Therefore, blood concentrations of TAC and CsA were also analyzed in addition to MPA and Mycophenolic acid β-D-glucuronide (MPAG).

### 2.1. Mycophenolic Acid

**Figure 1:**
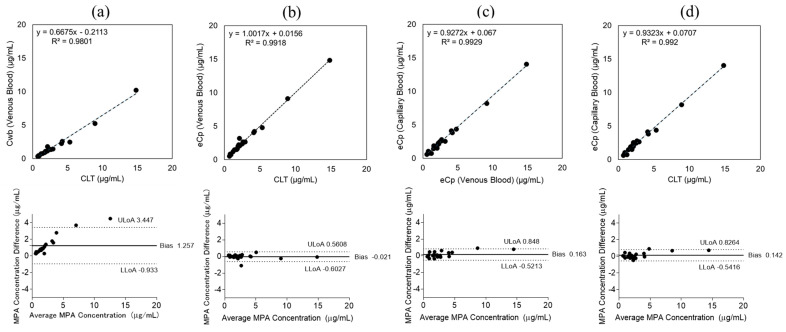
Mycophenolic acid (MPA) blood concentrations were measured in 21 patients. (**a**) Comparison of MPA concentrations measured by clinical laboratory testing (CLT) and the Microvolume method (MM). Linear regression and Bland–Altman analyses of venous blood MPA concentrations without hematocrit correction (y = 0.6675x − 0.2113, R^2^ = 0.9801; mean bias 1.257 μg/mL). (**b**) Comparison of MPA concentrations measured by CLT and MM after hematocrit correction (y = 1.0017x + 0.0156, R^2^ = 0.9918; mean bias −0.021 μg/mL). (**c**) Comparison of venous and capillary blood MPA concentrations measured by MM (y = 0.9272x + 0.067, R^2^ = 0.9929; mean bias 0.163 μg/mL). (**d**) Comparison of venous blood concentrations obtained by CLT and hematocrit-corrected capillary blood MPA concentrations measured by MM (y = 0.9323x + 0.0707, R^2^ = 0.992; mean bias 0.142 μg/mL). Axis labels used in these figures are summarized in Table 2.

Venous blood concentrations measured by clinical laboratory testing (CLT) and MM were compared to evaluate the validity of the microvolume assay. When uncorrected for hematocrit, simple linear regression analysis yielded the equation y = 0.6675x − 0.2113 with a coefficient of determination R^2^ = 0.9801 (Figure 1a). The Bland–Altman plot demonstrated a mean bias of 1.257 μg/mL (95% CI, −0.933 to +3.447). After hematocrit correction, regression analysis showed y = 1.0017x + 0.0156 with R^2^ = 0.9918, indicating excellent correlation (Figure 1b). Bland–Altman analysis showed a mean bias of −0.021 μg/mL (95% CI, −0.6027 to +0.5608), demonstrating reduced variability and strong agreement between CLT and MM.

To assess the validity of measurements obtained using different sampling methods, MPA concentrations in venous and capillary blood were compared using MM. Linear regression analysis showed y = 0.9272x + 0.067 with R^2^ = 0.9929 (Figure 1c), and the Bland–Altman plot demonstrated a mean bias of 0.163 μg/mL (95% CI, −0.5213 to +0.848), confirming excellent concordance between the two sampling methods.

Next, hematocrit-corrected capillary blood concentrations measured by MM were compared with venous blood concentrations obtained by CLT to evaluate whether fingerstick samples could serve as a practical alternative to conventional venous sampling. Regression analysis yielded y = 0.9323x + 0.0707 with R^2^ = 0.992 (Figure 1d). The Bland–Altman plot showed a mean bias of 0.1424 μg/mL (95% CI, −0.5416 to +0.8264), demonstrating high consistency between the two methods.

**Figure 2:**
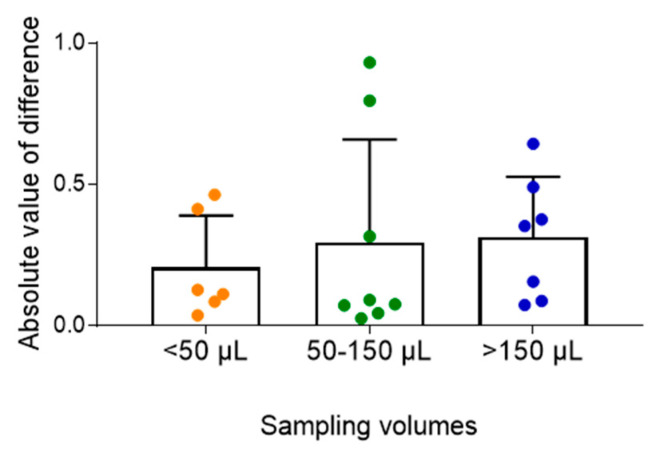
Effect of capillary blood sampling volume on mycophenolic acid (MPA) measurement accuracy. Absolute differences between venous and capillary MPA concentrations were consistent across sampling volumes (<50, 50–150, and >150 µL), indicating minimal volume-dependent variability.

To evaluate the effect of capillary sampling volume on measurement accuracy, absolute differences between venous and capillary blood concentrations (hematocrit-corrected) were analyzed across three sampling volume groups: <50 µL, 50–150 µL, and >150 µL. In all three groups, absolute differences between venous and capillary measurements remained small, suggesting that sampling volume did not significantly affect measurement accuracy (Figure 2).

### 2.2. Mycophenolic Acid β-D-Glucuronide

**Figure 3:**
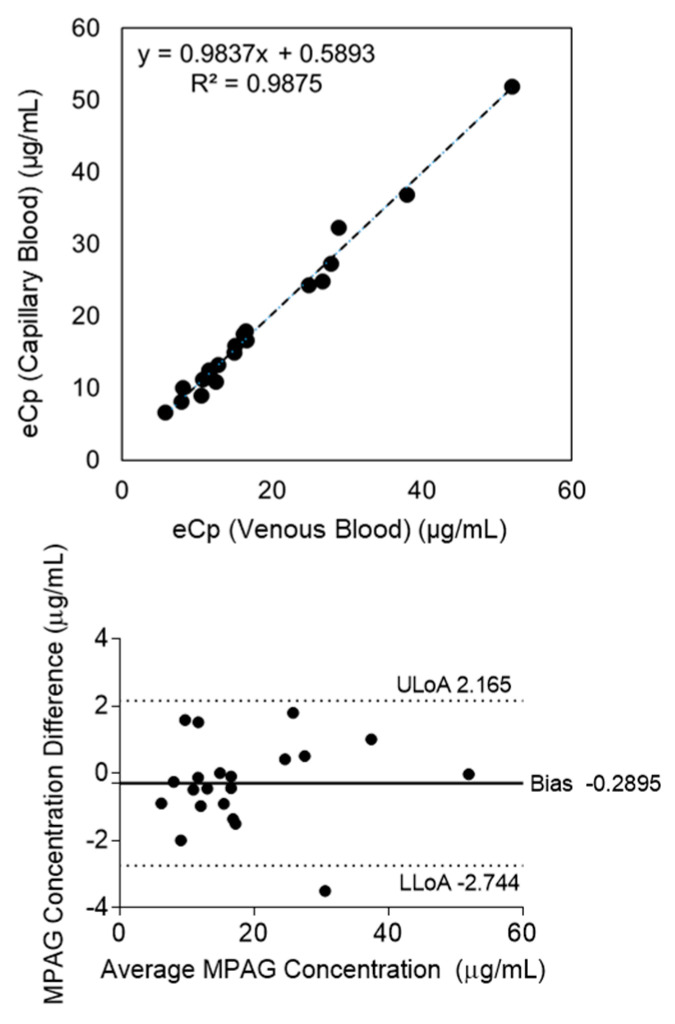
Mycophenolic acid β-D-glucuronide (MPAG) blood concentrations were measured in 21 patients. Comparison of MPAG concentrations in venous and capillary blood measured by the Microvolume method (MM). Linear regression analysis yielded y = 0.9837x + 0.5893 (R^2^ = 0.9875), and the Bland–Altman plot demonstrated a mean bias of −0.2895 μg/mL (95% CI, −2.744 to +2.165), indicating excellent agreement between the two sampling methods. Axis labels used in these figures are summarized in Table 2.

Because CLT was not available for MPAG, comparisons were made only between venous and capillary blood samples analyzed by MM. Regression analysis yielded y = 0.9837x + 0.5893 with R^2^ = 0.9875 (Figure 3), and the Bland–Altman plot demonstrated a mean bias of −0.2895 μg/mL (95% CI, −2.744 to +2.165), indicating excellent agreement between the two sampling methods.

**Figure 4:**
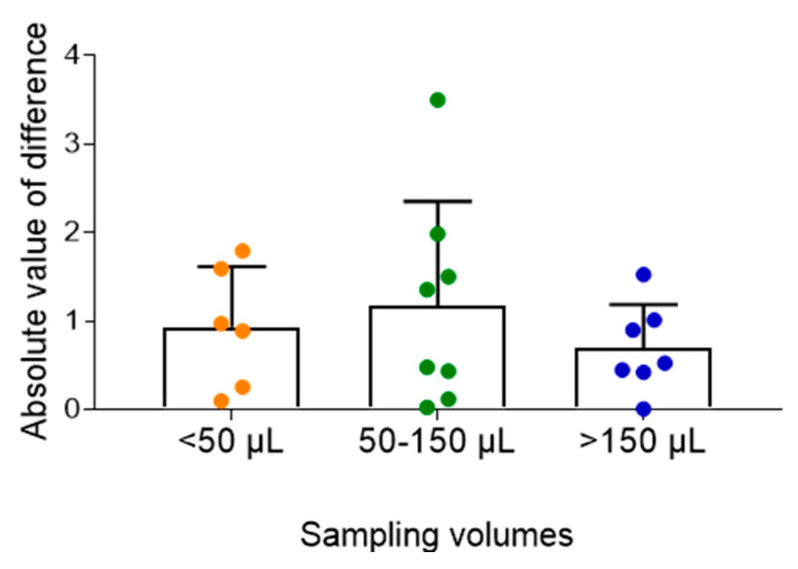
Effect of capillary blood sampling volume on mycophenolic acid β-D-glucuronide (MPAG) measurement accuracy. Absolute differences between venous and capillary MPAG concentrations were consistent across sampling volumes (<50, 50–150, and >150 µL), suggesting that sample volume had minimal impact on measurement accuracy.

The effect of capillary blood volume on MPAG measurement was also examined by dividing samples into three groups (<50 µL, 50–150 µL, and >150 µL). Absolute differences between venous and capillary blood concentrations showed no substantial variation across the three groups, suggesting that sample volume had minimal impact on the accuracy of MPAG measurements (Figure 4).

### 2.3. Tacrolimus

**Figure 5:**
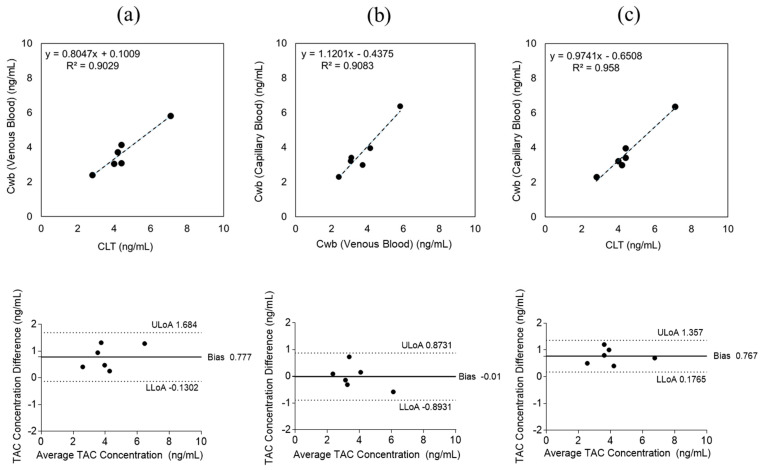
Tacrolimus (TAC) blood concentrations were measured in 6 patients. (**a**) Comparison of TAC concentrations measured by clinical laboratory testing (CLT) and the microvolume method (MM). Linear regression and Bland–Altman analyses of venous TAC concentrations measured by each method (y = 0.8047x + 0.1009, R^2^ = 0.9029; mean bias 0.777 ng/mL). (**b**) Comparison of venous and capillary TAC concentrations measured by MM (y = 1.1201x − 0.4375, R^2^ = 0.9083; mean bias −0.01 ng/mL). (**c**) Comparison of venous TAC concentrations obtained by CLT and capillary TAC concentrations measured by MM (y = 0.9741x − 0.6508, R^2^ = 0.958; mean bias 0.767 ng/mL). These results demonstrate excellent agreement between fingerstick and venous sampling. Axis labels used in these figures are summarized in Table 2.

Venous blood concentrations measured by CLT and MM were compared to assess the validity of the microvolume assay. Regression analysis yielded y = 0.8047x + 0.1009 with R^2^ = 0.9029 (Figure 5a), and the Bland–Altman plot indicated a mean bias of 0.777 ng/mL (95% CI, −0.1302 to +1.684), confirming a strong correlation between the two methods.

When comparing venous and capillary samples analyzed by MM, regression analysis showed y = 1.1201x − 0.4375 with R^2^ = 0.9083 (Figure 5b), and the Bland–Altman plot demonstrated a mean bias of −0.01 ng/mL (95% CI, −0.8931 to +0.8731), indicating strong agreement between sampling methods.

To determine whether fingerstick sampling could replace venous sampling for TAC measurement, capillary blood concentrations measured by MM were compared with venous concentrations obtained by CLT. Regression analysis yielded y = 0.9741x − 0.6508 with R^2^ = 0.958 (Figure 5c), and Bland–Altman analysis revealed a mean bias of 0.767 ng/mL (95% CI, +0.1765 to +1.357), demonstrating excellent concordance.

**Figure 6:**
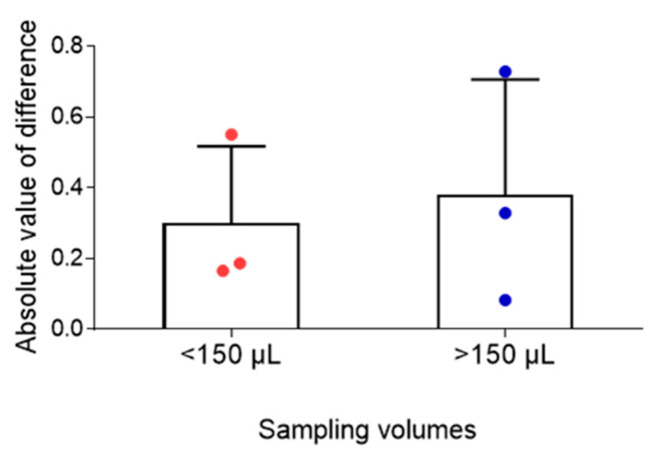
Effect of capillary blood sampling volume on tacrolimus (TAC) measurement accuracy. Absolute differences between venous and capillary TAC concentrations were consistent for both sampling volumes (<150 µL and ≥150 µL), indicating minimal impact of sampling volume on assay performance.

Evaluation of the influence of capillary sampling volume on TAC measurement showed that absolute differences between venous and capillary concentrations remained small across both sampling groups (<150 µL and ≥150 µL), suggesting minimal effect of sampling volume on assay performance (Figure 6).

### 2.4. Cyclosporine A

**Figure 7:**
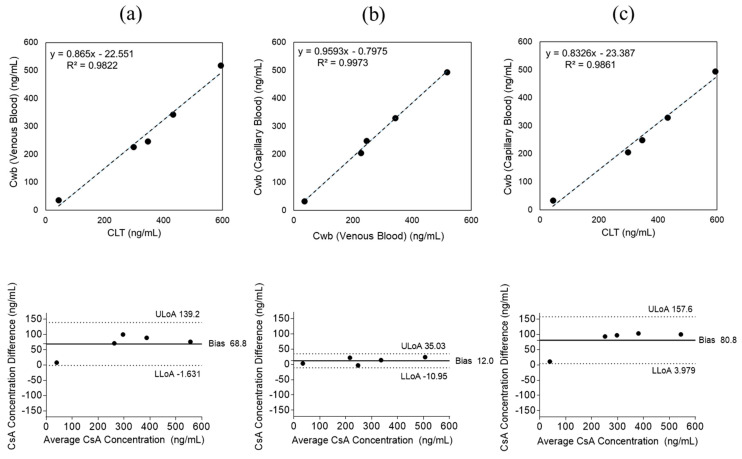
Cyclosporine A (CsA) blood concentrations were measured in 5 patients. (**a**) Linear regression and Bland–Altman analyses comparing venous CsA concentrations measured by clinical laboratory testing (CLT) and the microvolume method (MM) (y = 0.865x − 22.551, R^2^ = 0.9822; mean bias +68.8 ng/mL). (**b**) Comparison of venous and capillary CsA concentrations measured by MM (y = 0.9593x − 0.7975, R^2^ = 0.9973; mean bias +12.0 ng/mL). (**c**) Comparison of venous CsA concentrations obtained by CLT and capillary CsA concentrations measured by MM (y = 0.8326x − 23.387, R^2^ = 0.9861; mean bias +80.8 ng/mL), demonstrating strong consistency between fingerstick and venous sampling. Axis labels used in these figures are summarized in Table 2.

Venous blood concentrations obtained by CLT and MM were compared to assess the validity of the microvolume assay. Linear regression analysis yielded y = 0.865x − 22.551 with R^2^ = 0.9822 (Figure 7a), and the Bland–Altman plot indicated a mean bias of +68.8 ng/mL (95% CI, −1.631 to +139.2), demonstrating strong correlation between the two methods.

When comparing venous and capillary samples analyzed by MM, regression analysis yielded y = 0.9593x − 0.7975 with R^2^ = 0.9973 (Figure 7b), and the Bland–Altman plot showed a mean bias of +12.0 ng/mL (95% CI, −10.95 to +35.03), confirming excellent agreement.

Comparison between venous blood concentrations measured by CLT and capillary concentrations obtained by MM yielded y = 0.8326x − 23.387 with R^2^ = 0.9861 (Figure 7c).

**Table 2:** Summary of Sample Types, Analytical Methods, Blood Volume Requirements, and Figure Correspondence.

**Sampling Methods**	Venous Blood Sampling			Fingerstick Sampling
**Testing Methods**	Clinical Laboratory Testing (CLT)		Microvolume Method (MM)	
**Sample Types**	Whole Blood	Plasma	Whole Blood	Whole Blood
**Analyte**				
Mycophenolic acid (MPA)	–	liquid chromatography–tandem mass spectrometry (LC-MS/MS)	Newly Developed Simultaneous LC–MS/MS Quantification	
Tacrolimus (TAC)	Immunoassay	–		
Mycophenolic acid β-D-glucuronide (MPAG)	–			
Cyclosporine A (CsA)	Immunoassay	–		
**Required Blood Volume**	1 mL	1 mL	2.8 µL	2.8 µL
**Axis Labels Used in Figures**				
Figure 1		CLT	Whole-blood concentration (Cwb) (Venous Blood) Estimated plasma concentration (eCp) (Venous Blood)	eCp (Capillary Blood)
Figure 3			eCp (Venous Blood)	
Figure 5 and Figure 7	CLT		Cwb (Venous Blood)	Cwb (Capillary Blood)

Abbreviations used in Table 2 are defined as follows. CLT refers to Clinical Laboratory Testing, and MM indicates the Microvolume Method. MPA indicates mycophenolic acid, and MPAG represents mycophenolic acid β-D-glucuronide. TAC and CsA denote tacrolimus and cyclosporine A, respectively. LC–MS/MS is an abbreviation for liquid chromatography–tandem mass spectrometry, which is the core analytical method used in both approaches. In addition, Cwb represents whole-blood concentration, and eCp refers to estimated plasma concentration, both of which are used as axis labels in Figure 1, Figure 3, Figure 5 and Figure 7 to facilitate comparative interpretation.

The Bland–Altman plot demonstrated a mean bias of +80.8 ng/mL (95% CI, +3.979 to +157.6), showing strong consistency between the two methods.

**Figure 8:**
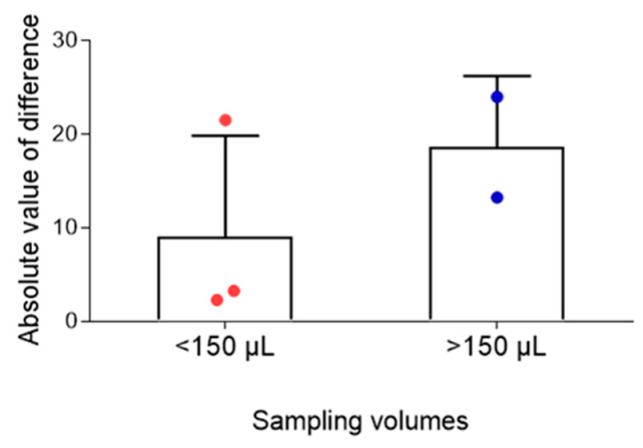
Effect of capillary blood sampling volume on cyclosporine A (CsA) measurement accuracy. Absolute differences between venous and capillary CsA concentrations were consistent across sampling volumes (<150 µL and ≥150 µL), indicating that sampling volume had minimal impact on measurement accuracy.

Analysis of the influence of capillary sampling volume showed that absolute differences between venous and capillary CsA concentrations were small in both sampling groups (<150 µL and ≥150 µL), indicating that sampling volume had no significant impact on measurement accuracy (Figure 8).

## 3. Discussion

In this study, we demonstrated the utility of MM for simultaneous quantification of MPA, MPAG, TAC, and CsA in pediatric patients. These results confirmed that multiple immunosuppressive drug concentrations can be reliably determined from a minimal volume of whole blood. Furthermore, we established that these four agents can be accurately quantified from fingerstick-derived capillary blood using the same method, highlighting the potential applicability of capillary microsampling for TDM in children.

Calcineurin inhibitors, such as TAC and CsA, distribute primarily in erythrocytes, whereas MPA and its metabolite MPAG are predominantly transported by plasma [1,13]. Previously, these pharmacokinetic differences necessitated separate analytical procedures for each drug, thereby increasing the complexity of laboratory workflows and the procedural burden on patients. The newly developed MM overcomes these limitations by enabling simultaneous quantification of all four agents in a 2.8 µL whole-blood sample [13]. Consistent with previous reports, our study in pediatric patients showed excellent correlations between MM and conventional CLT for MPA, TAC, and CsA [13]. Consistent with prior validation studies, TAC concentrations measured by immunoassay were consistently higher than those obtained by LC–MS/MS. This discrepancy reflects the known cross-reactivity of immunoassays with TAC metabolites and is attributable to assay-specific methodological characteristics rather than to limitations of capillary microsampling. While MPA is typically measured in plasma by conventional CLT, MM utilizes whole-blood samples. By applying hematocrit correction, plasma-equivalent concentrations obtained from MM were brought into even closer agreement with those measured by CLT, confirming the validity of the correction approach.

Given that accurate measurement can be achieved using minimal whole-blood volumes, this study underscores the potential of fingerstick sampling as an alternative to venipuncture, especially in pediatric settings, in which only limited blood volumes can be obtained. Fingerstick sampling offers several advantages, including procedural simplicity, reduced pain, and the feasibility of performing TDM in outpatient or even home settings [9]. These characteristics are particularly beneficial in children, for whom venous access is often difficult due to small vessel caliber and movement during sampling [2,15]. In comparisons between venous and capillary blood, concentrations of MPA, MPAG, TAC, and CsA showed excellent correlations and good agreement. These findings suggest that fingerstick samples may represent a feasible alternative to venous samples for these analytes under defined clinical conditions. Although previous studies employed different sampling and analytical methods, correlations between fingerstick and venous blood have been demonstrated in pediatric patients for MPA, TAC, and CsA [2,11,15]. Moreover, for MPA, TAC, and CsA, strong agreement was observed between capillary blood measured by MM (hematocrit-corrected for MPA) and venous blood measured by CLT. These results suggest that fingerstick-based capillary sampling analyzed with MM could serve as a practical alternative to conventional laboratory testing.

Previous studies have reported drug monitoring in pediatric patients using fingerstick samples, primarily employing VAMS or DBS techniques [2,11,15]. While VAMS offers advantages such as stability during storage [9], the capillary microsampling approach used in the present study provides faster turnaround times from collection to measurement, which is advantageous in clinical settings requiring prompt results. In fact, with the capillary microsampling method used in the present study, measurement results can be obtained in as little as 30 min after sample collection, whereas VAMS requires an additional drying period of approximately 2 h before analysis, thereby prolonging the overall processing time [10]. Additionally, capillary microsampling has the potential to allow for simultaneous measurement of hematological parameters, enabling concurrent evaluation of drug-related adverse effects such as cytopenia [16,17,18,19]. Thus, the method used in this study represents a clinically valuable approach complementary to existing microsampling techniques.

We also investigated whether the collected capillary blood volume affects the accuracy of concentration measurements. Previous hematological studies have suggested that smaller sample volumes may increase variability due to the influence of interstitial fluid contamination [20]. However, in our analyses of MPA and MPAG, absolute differences between hematocrit-corrected venous and capillary concentrations remained small across all three volume groups (<50 µL, 50–150 µL, and >150 µL). Similarly, for TAC and CsA, absolute differences were minimal in both sampling volume groups (<150 µL and ≥150 µL). These findings indicate that reliable measurements can be obtained even from very small sample volumes, underscoring the suitability of this approach for pediatric microsampling.

Finally, this study confirmed the feasibility of single-point measurement of MPA, TAC, and CsA from fingerstick blood using MM. Although AUC_0–12_-based assessments provide a more comprehensive evaluation of MPA exposure, particularly for individualized dosing, establishing the clinical utility of AUC_0–12_ estimation from fingerstick-based sampling remains an important subject for future investigation.

Although this study was industry-supported, study design, data analysis, and interpretation were conducted independently by the academic investigators. This study has several limitations. First, the sample size was relatively small, particularly for TAC and CsA, which limits the statistical power of agreement analyses. Second, clinical outcome concordance and dose adjustment impact were not evaluated in this study and warrant prospective investigation. In addition, intra-patient variability between sampling methods was not formally assessed, and repeated-measures concordance analyses would be required to more fully characterize analytical robustness in pediatric populations. Third, for MPA, hematocrit correction was performed using venous hematocrit values to ensure methodological consistency between capillary and venous comparisons in this validation study. This design enabled precise assessment of analytical agreement under controlled conditions. However, we acknowledge that fully decentralized fingerstick-only monitoring would require capillary-derived hematocrit estimation. Recent advances in microscale hematological technologies suggest that integration of capillary hematocrit assessment with microsampling-based LC–MS/MS analysis is technically feasible. Future studies should therefore evaluate the accuracy and practicality of capillary hematocrit determination in conjunction with microsampling-based TDM. Finally, multicenter validation in larger pediatric cohorts would further strengthen the generalizability of these findings.

Importantly, while previous work has demonstrated the feasibility of microvolume LC–MS/MS-based TDM in adult transplant recipients, the present study extends this concept to pediatric clinical practice, where sampling constraints, vascular access limitations, and patient burden differ fundamentally. These findings therefore support a translational framework in which analytically validated microsampling strategies may be adapted for all age groups while remaining tailored to population-specific clinical needs.

## 4. Materials and Methods

### 4.1. Study Population

From February 2025 to August 2025, we enrolled patients, aged 0–20 years, with kidney disease, including post-transplantation, lupus nephritis, and nephrotic syndrome, who were receiving mycophenolate mofetil (MMF) (Table 1). These patients attended the outpatient clinic of the Department of Pediatrics at Jichi Children’s Medical Center Tochigi, or were hospitalized, and provided written informed consent. At the time of clinically indicated blood sampling, capillary blood was collected from the fingertip concurrently with venous blood. No venipuncture was performed solely for the purpose of this study.

### 4.2. Capillary and Venous Blood Sampling

For capillary blood sampling, we used the multifunctional micro blood collection device, Capillary Cup^®^ (Japan Medical Leaf Co., Ltd., Tokyo, Japan), which contains dipotassium ethylenediaminetetraacetic acid (EDTA-2K) as an anticoagulant. The Capillary Cup^®^ consists of a detachable upper nozzle and a lower collection tube with a sealing cap. Both the inner surfaces of the nozzle and the collection tube are coated with EDTA-2K. The collection tube has a volume capacity of 200 μL [16]. Capillary blood was collected via fingerstick using Accu-Chek^®^ Safe-T-Pro Plus (Roche DC Japan Co., Ltd., Tokyo, Japan).

For venous blood collection, vacuum blood collection tubes containing either EDTA-2K or disodium ethylenediaminetetraacetic acid (EDTA-2Na) (Terumo Co., Ltd., Tokyo, Japan) were used, with a 23-gauge winged needle (Nipro Co., Ltd., Osaka, Japan) or a 24-gauge indwelling needle (Nippon Becton Dickinson Co., Ltd., Tokyo, Japan).

Blood sampling was performed by pediatricians, nurses, or laboratory technicians experienced in pediatric phlebotomy. All specimens were immediately frozen at −20 °C after collection. Capillary blood was obtained from the fingertip of the second, third, or fourth digit. After disinfecting the palmar surface of the fingertip with an alcohol swab, the hand was placed palm up, and the fingertip was punctured once with a lancet. Gentle pressure was applied around the puncture site, and 19–309 μL of blood was collected using the multifunctional micro-collection capillary cup. For venous sampling, 1 mL of blood was obtained by puncturing a peripheral vein of the extremities or the median cubital vein and was transferred into EDTA-2K and EDTA-2Na tubes. In addition, for patients receiving concomitant TAC or CsA, an additional 1 mL of blood was collected into an EDTA-2K tube.

### 4.3. Analytical Methods

Types of blood samples collected, measurement methods for immunosuppressants and metabolites, the required blood volume, and the axis labels used in the figures are summarized in Table 2. Concentrations of MPA were measured in both venous and capillary blood samples. For patients receiving concomitant TAC or CsA, blood concentrations were also determined. For quantification of MPA, venous blood samples were analyzed using two approaches. The first involved conventional CLT employing LC–MS/MS, which measures each analyte separately and generally requires a larger blood volume. The second MM utilized our recently developed LC–MS/MS technique, which differs from CLT in that it enables simultaneous quantification of multiple immunosuppressive agents from a minimal blood volume, including MPA, MPAG, TAC, and CsA, from an ultra-small volume (2.8 µL) of whole blood [13]. Briefly, 2.8 µL of whole blood were mixed with internal standards and extraction buffer, vortexed and centrifuged, and the supernatant was analyzed using LC–MS/MS for simultaneous quantification of MPA, MPAG, TAC, and CsA. Capillary blood samples were analyzed only by MM.

Table 2 summarizes analytical methods used in this study, including sample types, measurement approaches, and required blood volumes. This table serves as a reference for understanding comparisons presented in Figure 1, Figure 3, Figure 5 and Figure 7, and helps to clarify differences in analytical conditions across different experimental setups.

For CLT, MPA concentrations were determined exclusively in plasma rather than in whole blood. Plasma samples obtained from EDTA-2Na tubes were analyzed at an external facility (LSI Medience Co., Ltd., Tokyo, Japan) using an LC–MS/MS system (Waters Xevo TQ-S micro).

For MM, whole blood from venous samples collected in EDTA-2K tubes and capillary blood collected using an EDTA-2K-containing capillary cup were analyzed. Assays were performed using an LCMS-8050 triple quadrupole mass spectrometer equipped with an LC-30AD pump and SIL-30ACMP autosampler (Shimadzu Corporation, Kyoto, Japan) [13].

### 4.4. Hematocrit Correction

Quantitative measurements of MPA in whole blood were corrected for hematocrit, which was measured by routine CLT of venous blood samples. In the present validation study, venous hematocrit values were used to ensure methodological consistency between capillary and venous comparisons. Plasma-equivalent MPA concentrations were estimated using the following conversion formula:

(1) $$eCp=Cwb\times \frac{100}{100-Ht}$$

where eCp represents the estimated plasma concentration, Cwb is the measured whole-blood concentration, and Ht denotes the hematocrit value (%).

MPAG concentrations were measured by MM using whole blood from both venous (EDTA-2K tubes) and capillary samples.

For TAC and CsA, venous blood concentrations were measured using both CLT and MM, whereas capillary blood was analyzed only by MM. CLT was employed with whole blood collected in EDTA-2K tubes using an electrochemiluminescence immunoassay with a Cobas 8000 analyzer (Roche Diagnostics, Tokyo, Japan) in the hospital’s clinical laboratory. MM was conducted using whole blood obtained from venous samples collected in EDTA-2K tubes and from capillary blood collected with the EDTA-2K-containing capillary cup.

### 4.5. Statistical Analysis

To verify the validity of MM compared with CLT, linear regression analysis and Bland–Altman plots were used for venous samples of MPA, TAC, and CsA. For MPA, both hematocrit-uncorrected and corrected values were analyzed.

Furthermore, to evaluate the validity of capillary blood samples compared with those from venous blood, the same analyses (linear regression and Bland–Altman plots) were conducted for MPA, MPAG, TAC, and CsA using MM.

Additionally, to assess whether capillary blood could serve as an alternative to samples collected by CLT, results obtained from capillary blood measured by MM were compared with those from venous blood analyzed by CLT, using linear regression and Bland–Altman plots. For MPA, hematocrit-corrected values from capillary whole-blood samples were used for comparison.

To examine the influence of capillary sampling volume on concentration measurements, samples were arranged in ascending order of collected volume. For MPA and MPAG, data were divided into three groups, and for TAC and CsA, into two groups. Absolute differences between venous and capillary blood concentrations measured by MM were plotted for comparison.

All statistical analyses were performed using GraphPad Prism 7 for Windows (version 7.04; GraphPad Software, San Diego, CA, USA).

Agreement between methods was evaluated using simple linear regression and Bland–Altman analyses. Correlation strength was assessed using coefficients of determination (R^2^). Mean bias and 95% limits of agreement were calculated. Given the limited sample size, statistical analyses were considered exploratory in nature and intended to assess analytical concordance rather than to establish definitive clinical interchangeability.

This study was approved by the Clinical Research Ethics Review Committee of Jichi Medical University Hospital (approval number: 24-127). Written informed consent was obtained from both patients and their guardians. Because participants were children, consent was also obtained using age-appropriate documents containing the same information as the guardian consent form, written in language understandable to children.

## 5. Conclusions

In this study, we demonstrated that MM enables accurate and simultaneous quantification of MPA, TAC, and CsA from minimal whole-blood volumes obtained from pediatric patients. Furthermore, the combination of minimally invasive fingerstick microsampling and MM proved to be highly useful. This approach provides a practical and patient-friendly alternative to conventional venipuncture-based TDM. Because it allows for simultaneous measurement of multiple immunosuppressive agents with a very small sample volume, it is particularly advantageous in pediatric clinical practice, where blood collection is often challenging.

Moreover, this method shows great potential for use in outpatient and home-based TDM, offering a new clinical approach that reduces the physical and psychological burdens associated with traditional sampling, while supporting individualized and child-centered immunosuppressive therapy.

Future investigations should include larger multicenter validation studies, assessment of clinical decision concordance and intra-patient variability, and development of capillary-based hematocrit estimation strategies to enable fully decentralized home-based TDM.

Collectively, these findings establish a practical foundation for minimally invasive, patient-centered therapeutic drug monitoring in pediatric immunosuppressive therapy and represent an important step toward clinically implementable microvolume precision pharmacotherapy.

## Figures and Tables

**Table 1:** Patient characteristics.

	Age (Years)	Sex	Underlying Disease	MMF	TAC	CsA	Hematocrit (%)	BMI (kg/m^2^)
1	2	Male	SRNS	◯			44.9	18.2
2	3	Male	FRNS	◯			43.2	16.6
3	6	Male	FRNS	◯			47.0	25.6
4	7	Female	FRNS	◯			41.9	19.6
5	10	Male	FRNS	◯			38.7	17.2
6	10	Female	SRNS	◯		◯	39.5	17.9
7	11	Female	FRNS	◯			41.2	14.6
8	13	Female	Post-kidney transplantation (bilateral hypoplastic kidney)	◯	◯		31.0	16.3
9	14	Male	FRNS	◯			45.0	18.8
10	14	Male	Post-kidney transplantation (bilateral hypoplastic kidney)	◯	◯		47.5	20.3
11	14	Female	Lupus nephritis	◯			46.7	23.5
12	14	Male	SRNS	◯		◯	42.7	17.3
13	14	Male	Post-kidney transplantation (bilateral hypoplastic kidney)	◯	◯		39.5	18.5
14	15	Female	SRNS	◯			37.6	18.3
15	16	Male	SRNS	◯		◯	44.3	21.1
16	16	Male	Post-kidney transplantation (bilateral hypoplastic kidney)	◯	◯		38.2	26.9
17	17	Male	FRNS	◯			48.4	18.9
18	19	Female	FRNS	◯		◯	36.3	29.1
19	19	Male	SRNS	◯		◯	42.6	20.4
20	19	Female	SRNS	◯	◯		36.0	21.3
21	19	Male	Lupus nephritis	◯	◯		49.6	25.8

Abbreviations used in Table 1 are defined as follows. SRNS refers to steroid-resistant nephrotic syndrome, while FRNS indicates frequently relapsing nephrotic syndrome. MMF indicates mycophenolate mofetil, and TAC and CsA denote tacrolimus and cyclosporine A, respectively. BMI represents body mass index.

## Data Availability

The original contributions presented in this study are included in the article. Further inquiries can be directed to the corresponding author.

## Abbreviations

The following abbreviations are used in this manuscript:

TDM	Therapeutic Drug Monitoring
MPA	Mycophenolic Acid
TAC	Tacrolimus
CsA	Cyclosporine A
AUC_0–12_	Area Under the Concentration–Time Curve from 0 to 12 h
LC-MS/MS	Liquid Chromatography–Tandem Mass Spectrometry
EDTA-2K	Dipotassium Ethylenediaminetetraacetic Acid
EDTA-2Na	Disodium Ethylenediaminetetraacetic Acid
CLT	Clinical Laboratory Testing
MM	Microvolume Method
MPAG	Mycophenolic acid β-D-glucuronide
VAMS	Volumetric Absorptive Microsampling
DBS	Dried Blood Spot
